# Non-inferiority versus equivalence clinical trials in assessing biological products

**DOI:** 10.1590/S1516-31802011000300011

**Published:** 2011-05-05

**Authors:** Valdair Ferreira Pinto

**Affiliations:** MD. Independent Consultant in Pharmaceutical Medicine, São Paulo, Brazil.

Dear Editor,

Biological products currently represent the fastest growing and best performing sector of the pharmaceutical industry for two reasons: they succeed in addressing many unmet medical needs and are frequently marketed at premium prices. The patents of many biological products have either expired or are about to expire, thus creating interest in follow-on products, also known as biosimilars. Recently, the Brazilian regulatory agency Anvisa (Agência Nacional de Vigilância Sanitária) published new regulations for approval of new biological products and biosimilars.^1^ The development and approval of biosimilars is subject to a number of legal, regulatory, scientific and clinical challenges.

The World Health Organization (WHO) has issued a Guideline on Evaluation of Similar Biotherapeutic Products^2^ to provide worldwide principles for assessing similar biological products. This document is useful and consistent with the guidance from the European Medicines Agency (EMA). However, with regard to clinical evaluation, WHO clearly states that in principle, equivalence study designs shall be preferred, while non-inferiority study designs may be considered when appropriately justified. This recommendation is made under the assumption that greater efficacy may be associated with an increase in adverse events and that non-inferiority may contradict the principle of similarity. We are of the opinion that this position oversimplifies the issue and that there might be more justification for non-inferiority study designs than the document seems to suggest.

A conclusion of non-inferiority does not exclude a possible superiority of the tested treatment, but a demonstration of superiority in efficacy does not necessarily mean an increase in adverse events and, alternatively, a demonstration of equivalence in efficacy does not ensure equivalence in tolerability. Usually, efficacy clinical trials are not powered to detect differences in the frequency of adverse events. The ideal situation would be to design the study as a non-inferiority study for efficacy and for superiority of safety, thereby ensuring power for both objectives. Sample sizes have to be calculated for each objective and then the maximum of the two values should be chosen. However, this approach raises issues and problems and it is not easily implementable.^3^ Moreover, primary endpoints are seldom defined as direct measurements of pharmacodynamic effects. For example, in the clinical assessment of insulin, the primary endpoint is the reduction in HbA1c (glycated hemoglobin) and not glycemia; similarly, in the clinical assessment of low-molecular-weight heparins, the recommended primary endpoint is the reduction of thromboembolic events and not direct measurements of anticoagulation. In situations in which endpoints are defined as measurements of morbidity or mortality, findings of superiority are not necessarily unwanted. On the other hand, adverse events should be assessed separately with valid comparisons.

Although non-inferiority clinical trials are more complex than the traditional superiority studies, they usually require 20-30% fewer patients than the corresponding equivalence trials.^4^ Therefore, while making decisions between equivalence and non-inferiority designs, several factors should be considered, such as the impact of superiority on the primary endpoint, validity of comparisons of frequencies of adverse events and, importantly, cost and ethical issues associated with larger sample sizes. All factors considered, it is likely that non-inferiority designs might be preferred more often than equivalence designs.
